# Supplementary material for: Inhibition of mutant RAS-RAF interaction by mimicking structural and dynamic properties of phosphorylated RAS
Source: eLife. 2022 Dec 2;11:e79747. doi: 10.7554/eLife.79747 (PMC9762712; doi:10.7554/eLife.79747)
Supplement: Supplementary file 1. — a The average number of water molecules within 5 Å of GTP were calculated over the course of the ligand-bound HRASG12D systems. b Total simulation time performed for ligand- HRASG12D complexes and changes in the backbone RMSF profiles of cerubidine-, tranilast-, nilotinib-, and epirubicin-bound HRASG12D systems with respect to those of HRASG12D. c The results of PRS calculations for the transition between initial and target states. [file elife-79747-supp1.zip › RAS_woHL_v2SI.pdf]

Supplementary Information: Inhibition of mutant RAS-RAF interaction by mimicking structural and dynamic properties of phosphorylated RAS

Metehan Ilter[1,†,‡], **Ramazan Kaşmer**[2,3,‡], **Farzaneh Jalalypour**[4,§], **Canan Atilgan**[4], **Ozan Topcu**[2], **Nihal Karakaş**[2,5,*], **Ozge Sensoy**[6,7,*]

***For correspondence:** nkarakas@medipol.edu.tr (NK); osensoy@medipol.edu.tr (OS)

[‡]These authors contributed equally to this work

Present address: [†]Molecular Simulations and Design Group, Max Planck Institute for Dynamics of Complex Technical Systems, Magdeburg, Germany; [§]Department of Applied Physics, Science for Life Laboratory, KTH Royal Institute of Technology, Stockholm, Sweden

[1]Graduate School of Engineering and Natural Sciences, Istanbul Medipol University, Istanbul, Turkey; [2]Cancer Research Center, Institute for Health Sciences and Technologies (SABITA), İstanbul Medipol University, İstanbul, Tukey; [3]Medical Biology and Genetics Program, Graduate School for Health Sciences, Istanbul Medipol University, Istanbul Turkey; [4]Faculty of Engineering and Natural Sciences, Sabanci University, Istanbul, Turkey ; [5]Department of Medical Biology, School of Medicine, Istanbul Medipol University, Istanbul, Turkey ; [6]Department of Computer Engineering, School of Engineering and Natural Sciences, Istanbul Medipol University, Istanbul, Turkey; [7]Regenerative and Restorative Medicine Research Center (REMER), Institute for Health Sciences and Technologies (SABITA), İstanbul Medipol University, İstanbul. Turkey

Supplementary file 1a The average number of water molecules within 5 Å of GTP were calculated over the course of the ligand-bound HRAS^{G12D} systems.

Ligand-bound H-RAS^{G12D}	μ_{water}
Cerubidine	119.1±0.3
Tranilast	104.5 ±0.4
Nilotinib	95.3 ±0.5
Epirubicin	106.2 ±0.4

Supplementary file 1b Total simulation time performed for ligand- HRAS^{G12D} complexes and changes in the backbone RMSF profiles of cerubidine-, tranilast-, nilotinib-, and epirubicin-bound HRAS^{G12D} systems with respect to those of HRAS^{G12D}.

Ligand	Duration (ns)	ΔRMSF(Y32) (Å)	$\mu_{\Delta RMSF(RAF\text{-}RBD)}$ (Å)	$\mu_{\Delta RMSF(RAF\text{-}CRD)}$ (Å)
Cerubidine	3069	1.0 ± 0.4	1.1 ± 0.6	0.2 ± 0.1
Tranilast	3705	1.0 ± 0.6	0.9 ± 0.7	0.3 ± 0.2
Nilotinib	3053	1.4 ± 0.7	1.5 ± 0.8	0.3 ± 0.2
Epirubicin	2884	2.1 ± 0.6	1.6 ± 0.9	0.2 ± 0.2

Supplementary file 1c The results of PRS calculations for the transition between initial and target states.

Ligand	State	D12-P34 (Å)	G60-GTP (Å)	PRS selected residues	PRS overlap*(O^i)*
Nilotinib	Target state-1	22.4 (open)	5.1 (closed)	34, 35, 33, 37, 32, 36	0.70-0.62
	Target state-2	15.3 (partially open)	12.7 (open)	61, 62, 63, 23, 22, 65	0.57-0.51
	Target state-3	22.6 (open)	19.8 (open)	34, 35, 33, 37, 36, 66	0.66-0.59
Tranilast	Target state-1	27.0 (open)	9.1 (closed)	32, 36, 33, 37, 34, 35	0.74-0.67
	Target state-2	14.8 (partially opened)	12.3 (open)	22, 18, 23, 104, 87, 6	0.55-0.54
	Target state-3	20.1 (open)	14.8 (open)	34, 33, 35, 32, 37	0.54-0.50
Epirubicin	Target state-1	29.0 (open)	7.7 (closed)	32, 33, 34, 35, 37, 36	0.61-0.51
	Target state-2	13.3 (partially opened)	17.8 (open)	63,62	0.51-0.50
	Target state-3	22.0 (open)	15.2 (open)	34, 35, 33, 37, 36, 66	0.61-0.59
