# Supplementary figures and images for: Inhibition of mutant RAS-RAF interaction by mimicking structural and dynamic properties of phosphorylated RAS
Source: eLife. 2022 Dec 2;11:e79747. doi: 10.7554/eLife.79747 (PMC9762712; doi:10.7554/eLife.79747)

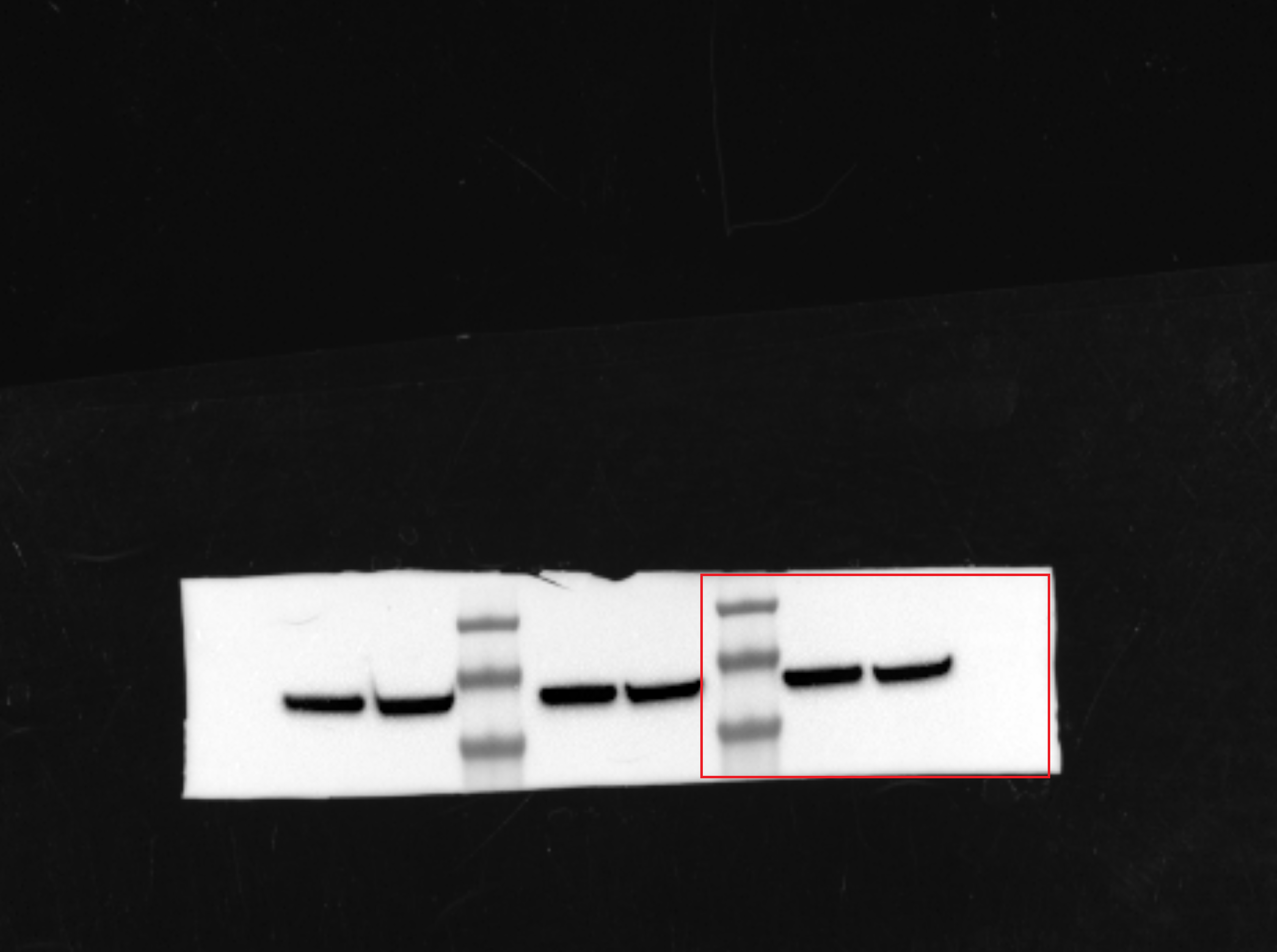

Supplement: Source data 1. [file elife-79747-data1.zip › (1)/Figure 10 (e) Anti Bactin.tif]

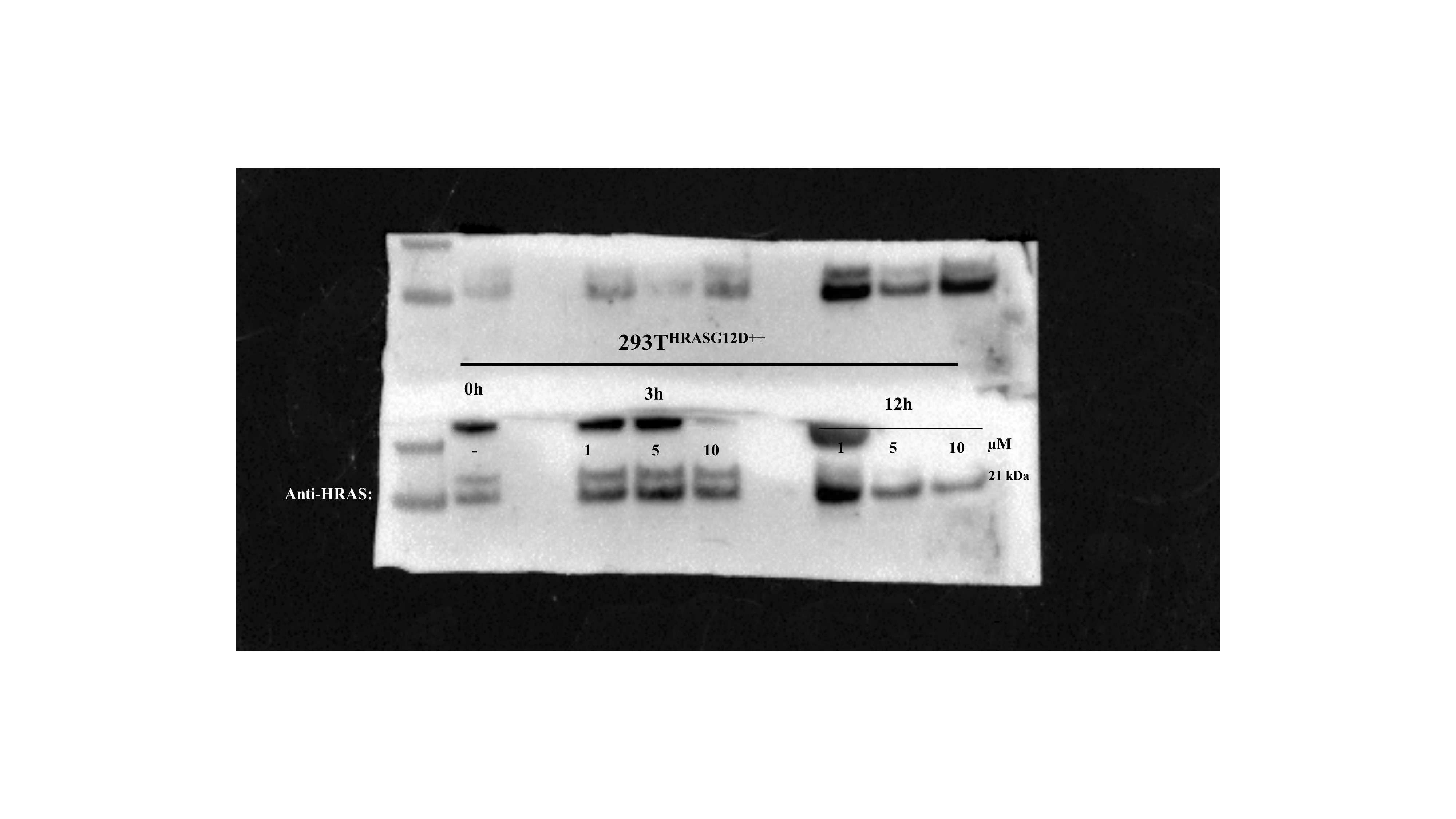

Supplement: Source data 2. [file elife-79747-data2.zip › (2)/Figure 12 (e) anti HRAS.JPG]

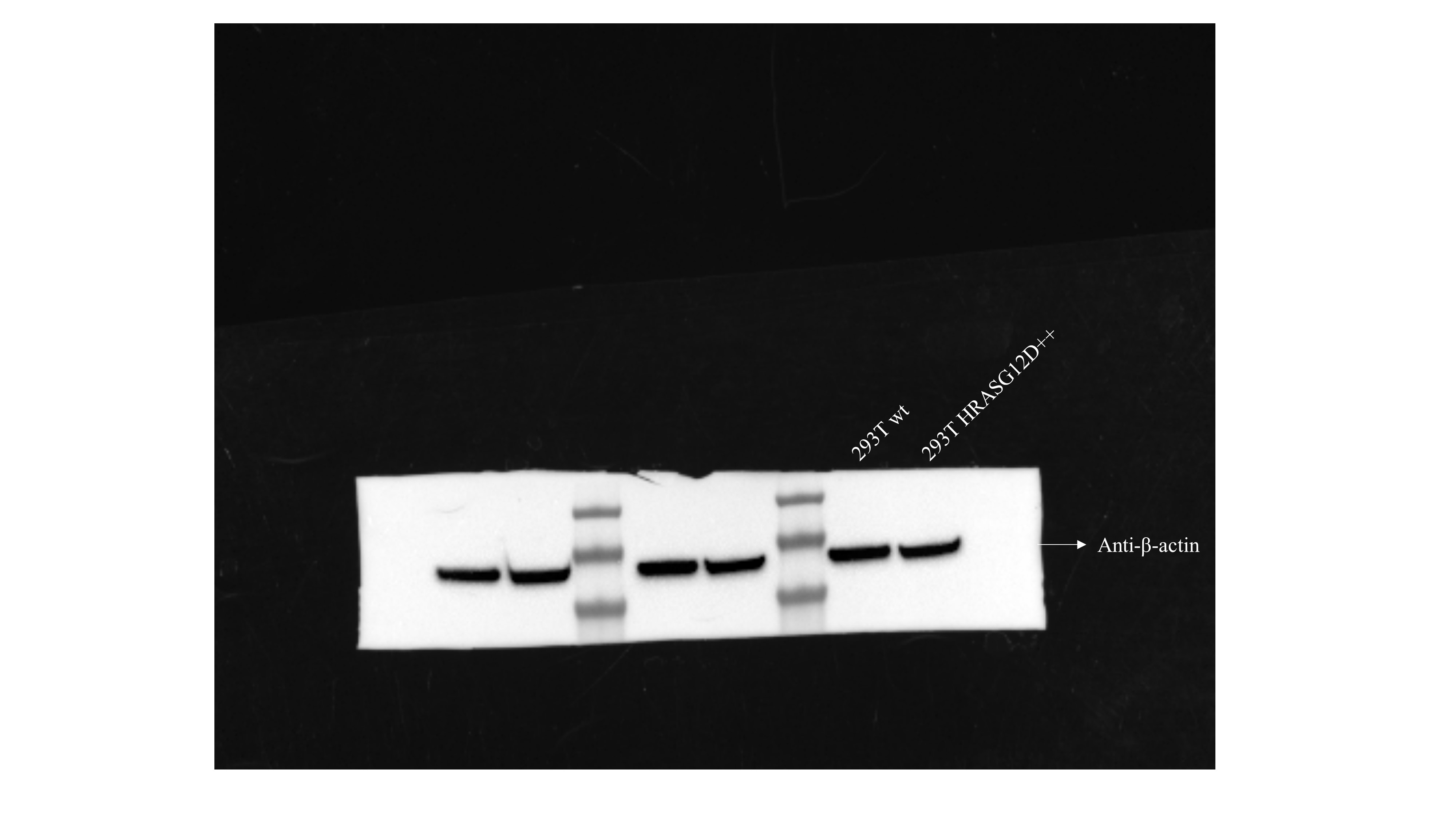

Supplement: Source data 2. [file elife-79747-data2.zip › (2)/Figure 10 (e) Anti Bactin.JPG]

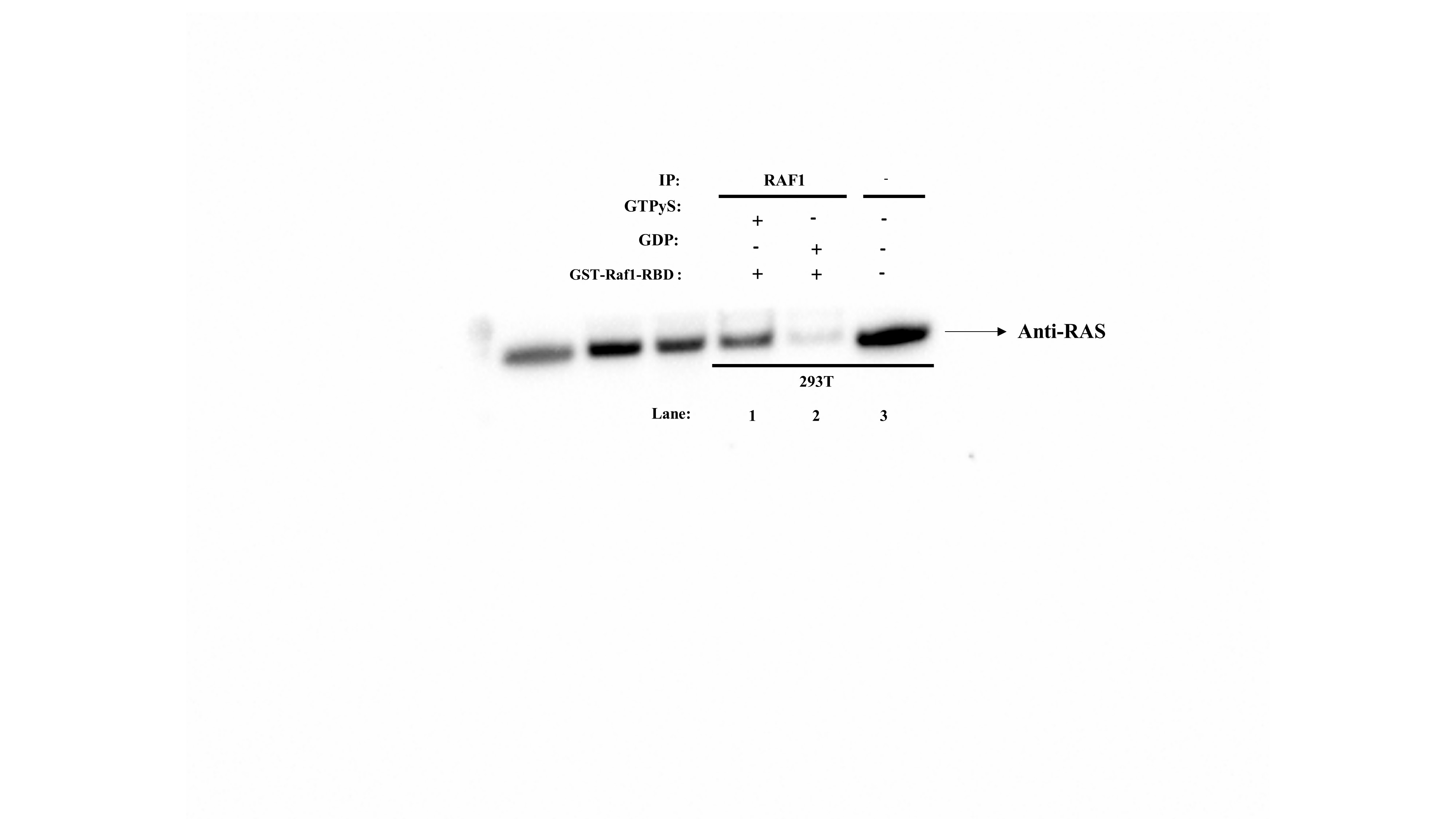

Supplement: Source data 2. [file elife-79747-data2.zip › (2)/Figure 12 (b).JPG]

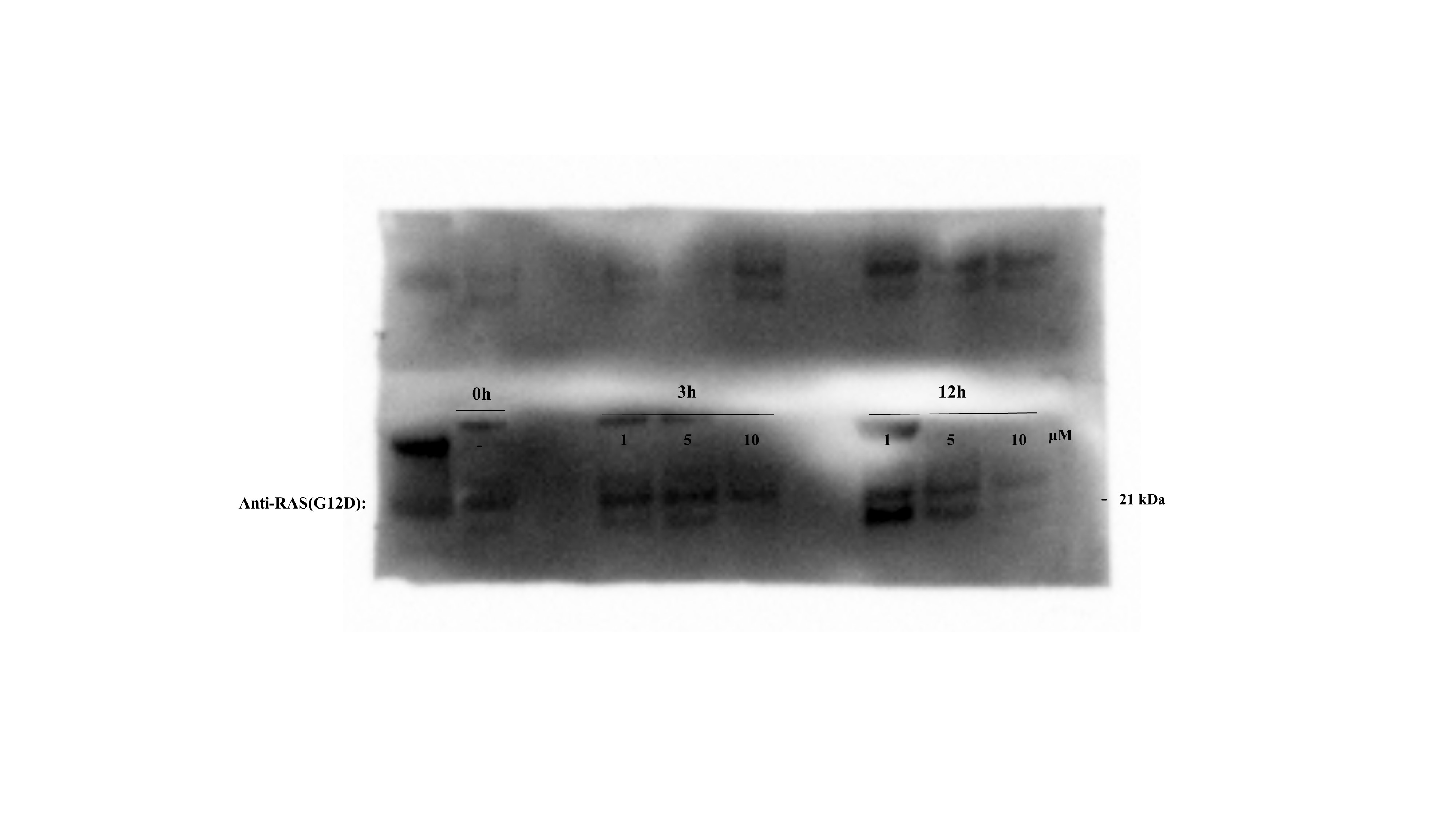

Supplement: Source data 2. [file elife-79747-data2.zip › (2)/Figure 12 (e) anti RASG12D.JPG]

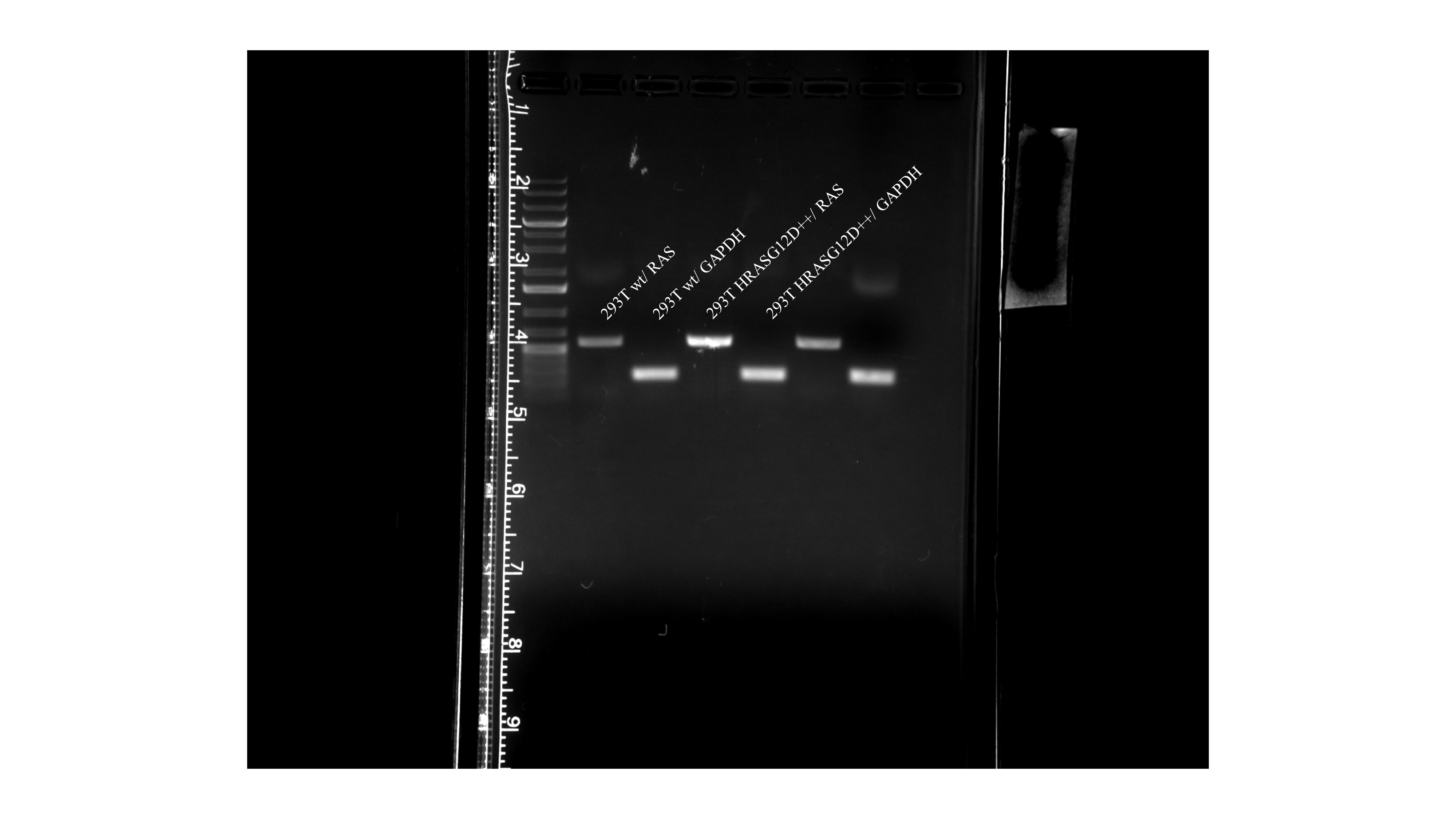

Supplement: Source data 2. [file elife-79747-data2.zip › (2)/Figure 10 (c).JPG]

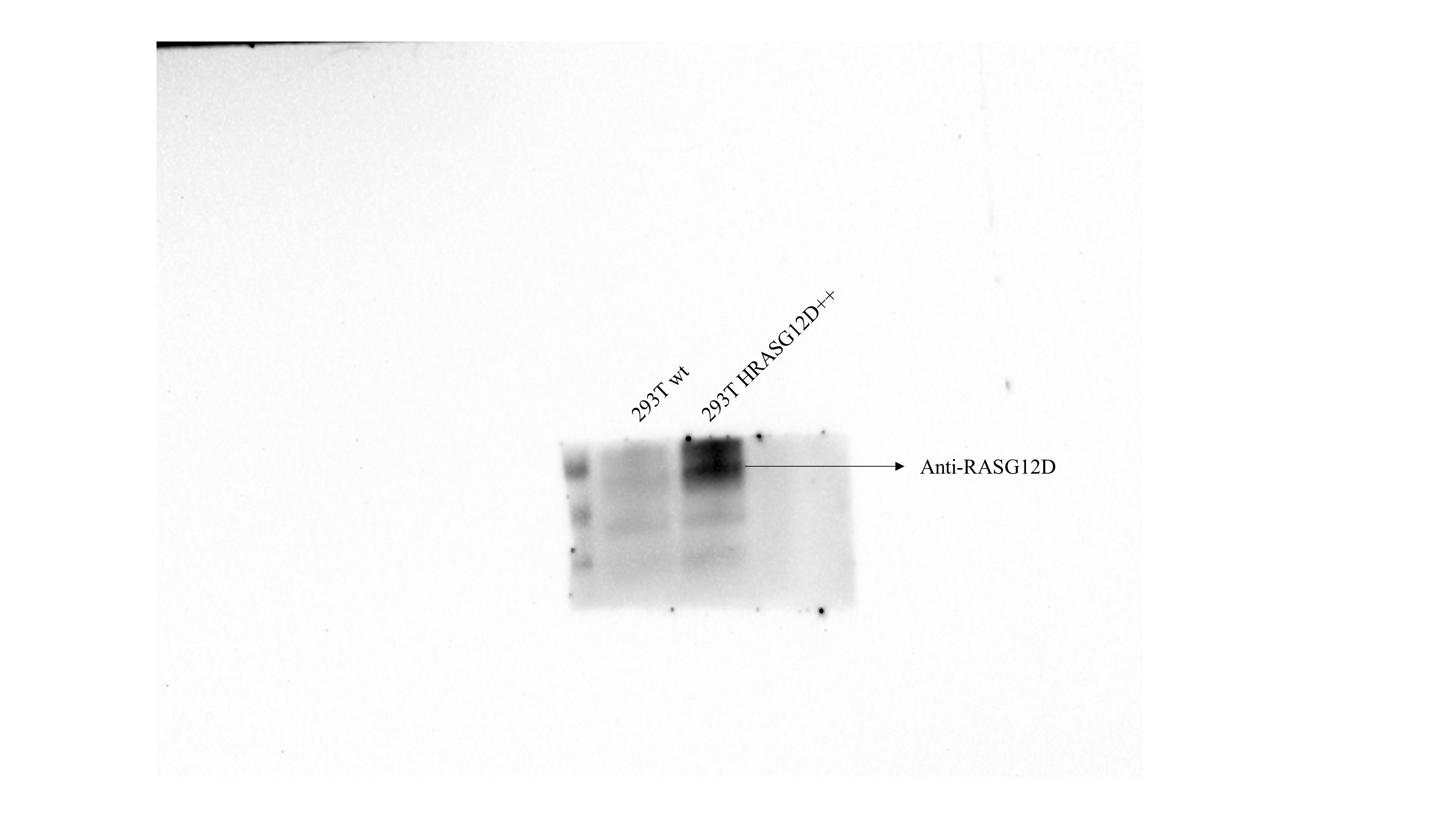

Supplement: Source data 2. [file elife-79747-data2.zip › (2)/Figure 10 (e) Anti RASG12D.JPG]
